# Changes in oscillatory patterns of microstate sequence in patients with first-episode psychosis

**DOI:** 10.1038/s41597-023-02892-8

**Published:** 2024-01-05

**Authors:** Dong-Dong Zhou, Hong-Zhi Li, Wo Wang, Li Kuang

**Affiliations:** 1 Mental Health Center, University-Town Hospital of Chongqing Medical University, Chongqing, China; 2 Department of Psychiatry, The First Affiliated Hospital of Chongqing Medical University, Chongqing, China

**Keywords:** Diagnostic markers, Psychosis, Data mining

## Abstract

We aimed to utilize chaos game representation (CGR) for the investigation of microstate sequences and explore its potential as neurobiomarkers for psychiatric disorders. We applied our proposed method to a public dataset including 82 patients with first-episode psychosis (FEP) and 61 control subjects. Two time series were constructed: one using the microstate spacing distance in CGR and the other using complex numbers representing the microstate coordinates in CGR. Power spectral features of both time series and frequency matrix CGR (FCGR) were compared between groups and employed in a machine learning application. The four canonical microstates (A, B, C, and D) were identified using both shared and separate templates. Our results showed the microstate oscillatory pattern exhibited alterations in the FEP group. Using oscillatory features improved machine learning performance compared with classical features and FCGR. This study opens up new avenues for exploring the use of CGR in analyzing EEG microstate sequences. Features derived from microstate sequence CGR offer fine-grained neurobiomarkers for psychiatric disorders.

## Introduction

Electroencephalography (EEG) is a convenient and noninvasive tool for recording brain electrical activity and is widely used in clinical practice and scientific research. A major advantage of EEG is its high temporal resolution, which allows for the investigation of brain activity at the millisecond level. Multichannel EEG can be clustered into several discrete scalp topographies, called microstates, which are quasistable for 80-120 ms^1^. Classical microstates include microstate A, B, C, and D, which together explain 65-84% of the global signal variance^2^. Worldwide researchers have consistently reported similar microstates to these four classical microstates, which also have high test-retest consistency^3^. EEG microstates are closely associated with resting functional connectivity derived from functional magnetic resonance imaging (fMRI), indicating that EEG microstates may reflect underlying synchronous neural activity to form large-scale brain networks^2^.

Microstates occurring over time form a microstate sequence. Given the evidence that a microstate reflects large-scale brain networks, it is reasonable to infer that microstate sequences reflect dynamic changes among different brain networks. Moreover, the properties derived from EEG microstate sequences are nearly independent of the various clustering algorithms used^4^, suggesting that we could derive stable biomarkers from microstate sequences. Thus, studying the characteristics of EEG microstate sequences could help us better understand human brain chronnectome features at a high temporal resolution.

Currently, there are several analytic approaches for microstate sequences. Many previous studies have reported the transition probabilities among different microstates; additionally, several significant differences in microstate transition probabilities have been found between normal controls and patients with psychiatric disorders^5,6^. However, it is possible that EEG microstate sequences cannot be modelled by a memoryless Markovian process and have a long-range correlation with a Hurst exponent larger than 0.5^7,8^. Entropy analyses showed that the sample entropy of the microstate sequence decreases as the template length increases in healthy subjects, but this has not been observed in patients with early-course psychosis^9^. The lower sample entropy suggests that there is some regularity in the microstate sequence of healthy subjects, but this regularity is absent in patients^9^. This view that there is regularity in microstate sequences was supported by another study that showed a recurrent neural network (RNN) with long short-term memory (LSTM) could accurately predict microstate sequences within 400 ms, but the accuracy dropped dramatically beyond 400 ms^10^. Li *et al*. (2020) developed a novel approach to perform microstate spectral analysis, utilizing multivariate empirical mode decomposition and the Hilbert-Huang transform, and revealed that these spectral features could be used to evaluate an individual’s level of consciousness^11^. However, this spectral analysis is directly based on multichannel EEG rather than microstate sequences, and the frequency domain properties of microstate sequences have not yet been characterized.

Chaos game representation (CGR), an iterated function system, can map a sequence of several discrete states to two-dimensional space, facilitating visualization of that sequence^12^. Analyses based on CGR have been widely used with deoxyribonucleic acid (DNA) and protein sequences and are regarded as a milestone in the development of graphical bioinformatics^13^. Considering the similarity of DNA and microstate sequences, with both consisting of four discrete states (A, T, C, G for DNA and A, B, C, D for microstate sequence), it naturally follows that the CGR approach can be applied to the microstate sequence. CGR can uniquely represent a sequence^13^; this property may enable us to construct chronnectome fingerprinting based on the CGR of the microstate sequence. Moreover, the frequency matrix CGR (FCGR) has been previously used as a feature for DNA or protein classification^13,14^. FCGR refers to counting the number of points on a predefined grid in CGR. Elements in each cell of the FCGR represent the frequency of subsequences. Naturally, the FCGR of microstate sequences may enable us to investigate the characteristics of subsequences within microstate sequences and be used as features for machine learning to distinguish neuropsychiatric patients and normal controls. Additionally, the properties of microstate sequences in the frequency domain remain unclear, with most previous studies having focused on the time domain characteristics of microstate sequences. Although CGR has been used for DNA or protein sequence analyses, no study has investigated the characteristics of microstate sequences based on CGR.

**Fig. 1:**
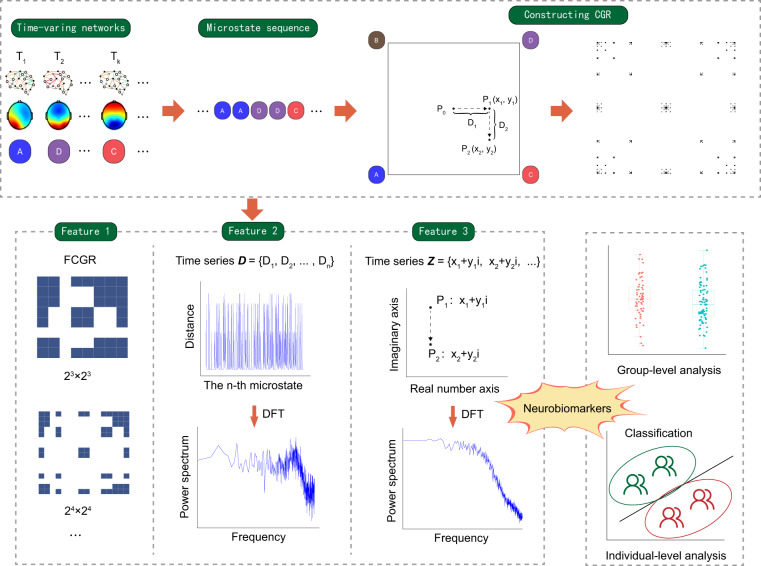
**An illustration of neurobiomarkers derived from microstate CGR features**. CGR, chaos game representation; FCGR, frequency matrix CGR. DFT, Discrete Fourier Transform.

Therefore, we proposed that microstate features derived from CGR might serve as neurobiomarkers at both the group and individual levels for psychiatric disorders (Fig. 1). The primary aim of this study was to explore the use of CGR in analyzing microstate sequences and to determine the effectiveness of CGR-derived features as potential neurobiomarkers.

## Results

### Traditional analyses

**Fig. 2:**
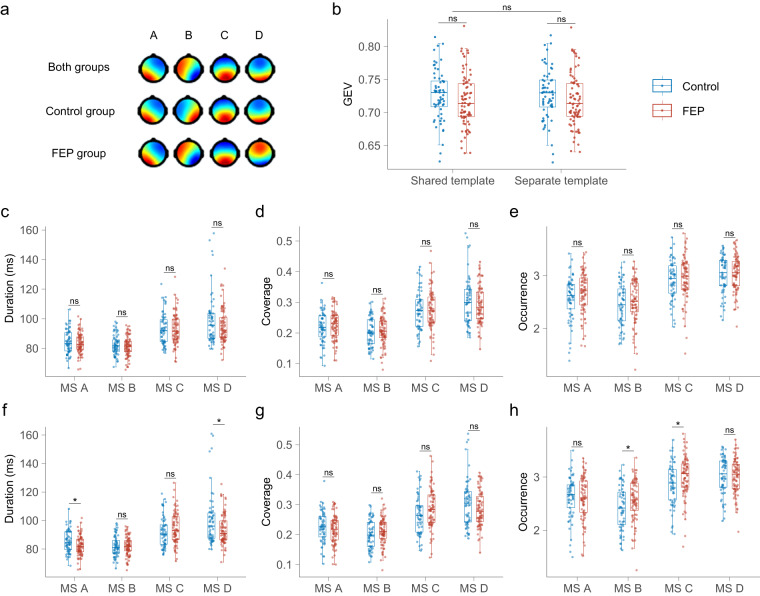
**Classical dynamic characteristics of microstates**. (**a**) The four canonical microstates in the shared template and separate template. (**b**) GEVs in each group for the shared template and separate template. Using the shared template: (**c**) duration of microstates in the control and FEP groups. (**d**) coverage of microstates in the control and FEP groups. (**e**) occurrence of microstates in the control and FEP groups. Using the separate template: (**f**) duration of microstates in the control and FEP groups. (**g**) coverage of microstates in the control and FEP groups. (**h**) occurrence of microstates in the control and FEP groups. ns, non-significant; GEV, global explained variance; FEP, first-episode psychosis. *p < 0.05.

The EEG data of ‘sub-2356A’ were removed because of an extreme outlier. Consequently, the study included 142 subjects in the subsequent analyses, comprising 61 healthy controls and 81 patients with first-episode psychosis (FEP). The four canonical microstates, labeled A, B, C, and D, were consistently identified across all templates and in all 100 iterations of microstate clustering (Fig. 2a and Supplementary Figures 1–3). The global explained variance (GEV) stood at 72.7% for the control group and 71.8% for the FEP group, consistent across both shared and separate templates (Fig. 2b).

When using a shared template, no significant differences were observed between the control and FEP groups in terms of mean duration, coverage, occurrence, and transition probability for any of the microstates (Fig. 2c–e, and Supplementary Table 1). However, with a separate template, the durations of microstates A and D were significantly longer in the control group compared to the FEP group. Conversely, microstates B and C occurred more frequently in the FEP group. No significant differences in microstate coverage were noted (see Fig. 2f–h). Transitions from microstate A to D and vice versa were significantly more frequent in the control group, whereas transitions between microstates B and C were more prevalent in the FEP group (as detailed in Supplementary Table 1).

### FCGR analyses

**Fig. 3:**
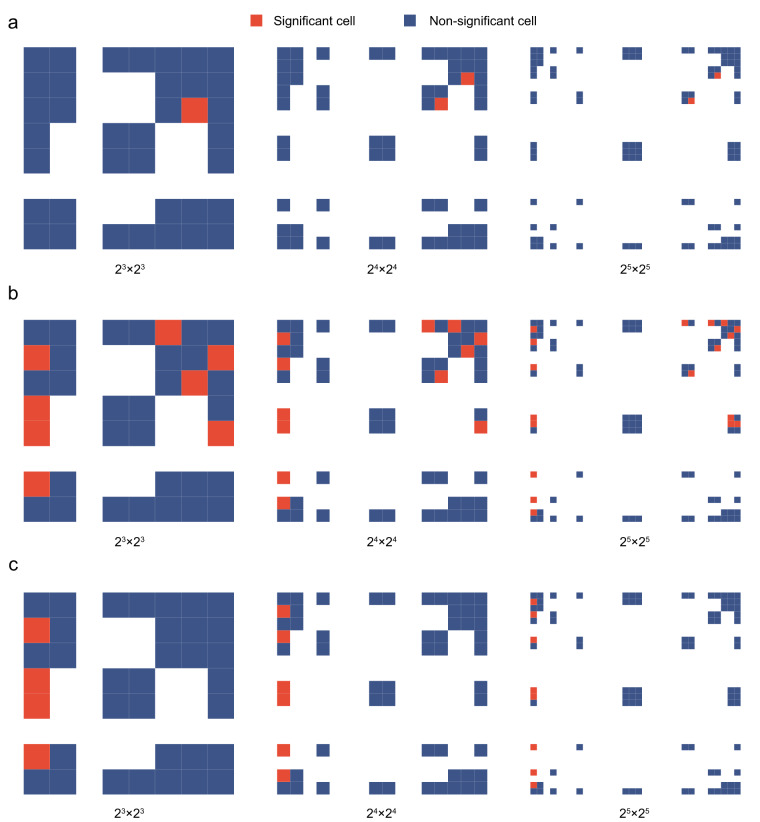
**Variation in cells of the FCGR at different resolutions between groups**. (**a**) variations in FCGR cells using the shared template. (**b**) variations in FCGR cells using the separate template. (**c**) variations in FCGR cells after FDR corrections using the separate template. CGR, chaos game representation; FCGR, frequency matrix CGR. FDR, false discovery rate.

Using the shared template revealed several cells in the FCGR that differed significantly between the FEP and control groups at resolutions of ${2}^{3}\times {2}^{3}$, ${2}^{4}\times {2}^{4}$, ${2}^{5}\times {2}^{5}$, ${2}^{6}\times {2}^{6}$, ${2}^{7}\times {2}^{7}$, and ${2}^{8}\times {2}^{8}$ (Fig. 3a). However, only the FCGR plots for the first three resolutions are shown in Fig. 3. This is because plots with higher resolutions become overly pixelated, leading to poor visualization. However, none of these results remained significant after adjusting for the false discovery rate (FDR). In contrast, using separate templates, a greater number of cells in the FCGR showed significant differences between the groups, with several cells maintaining their significance even after FDR correction (Fig. 3b, c).

### Analyses for time series ${\boldsymbol{D}}$

**Fig. 4:**
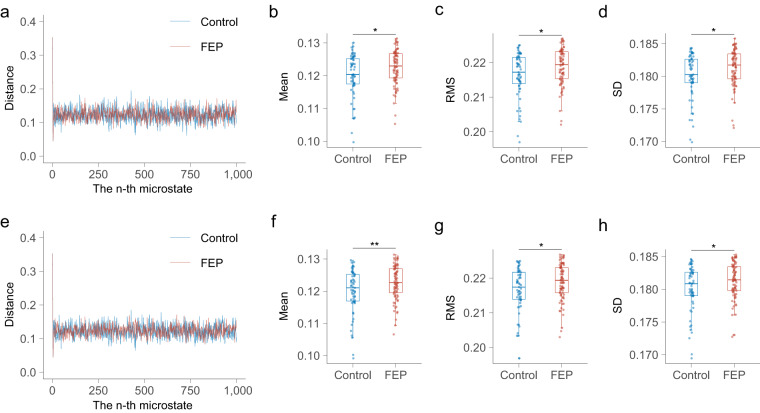
**The time domain characteristics of the time series** ***D***. Using the shared template: (**a**) mean distance of the first 1000 microstates in CGR in each group, with the Euclidean distance from the previous microstate to the current microstate on the vertical axis. (**b**) comparison of the mean distance between groups. (**c**) comparison of the RMS between groups. (**d**) comparison of the SD between groups. Using the separate template: (**e**) mean distance of the first 1000 microstates in CGR in each group, with the Euclidean distance from the previous microstate to the current microstate on the vertical axis. (**f**) comparison of the mean distance between groups. (**g**) comparison of the RMS between groups. (**h**) comparison of the SD between groups. FEP, first-episode psychosis; SD, standard deviation; RMS, root mean square.

**Fig. 5:**
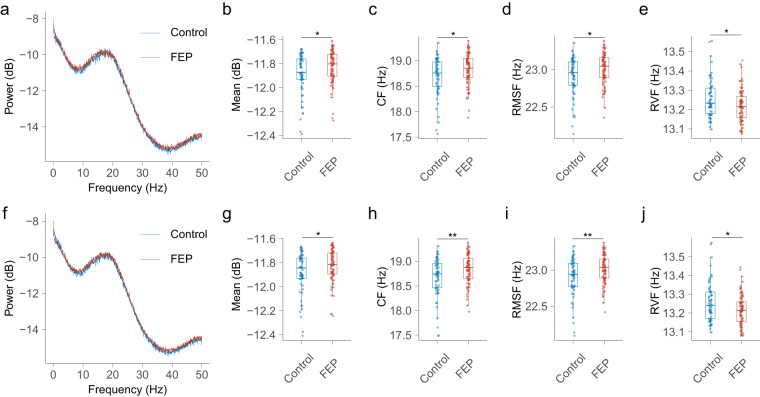
**The frequency domain characteristics of the time series** ***D***. Using the shared template: (**a**) the power spectrum of time series ${\boldsymbol{D}}$ in control and FEP groups. (**b**) comparison of the mean power between groups. (**c**) comparison of the CF between groups. (**d**) comparison of the RMSF between groups. (**e**). comparison of the RVF between groups. Using the separate template: (**f**). the power spectrum of time series ${\boldsymbol{D}}$ in control and FEP groups. (**g**). comparison of the mean power between groups. (**h**) comparison of the CF between groups. (**i**) comparison of the RMSF between groups. (**j**) comparison of the RVF between groups. FEP, first-episode psychosis; CF, centre of frequency; RMSF, root mean square frequency; RVF, root of variance frequency.

Using the shared template, the mean, standard deviation (SD), and root mean square (RMS) of the time series ${\boldsymbol{D}}$ in the control group were significantly lower than those in the FEP group (Fig. 4a–d). Regarding the frequency domain features, the mean power, centre of frequency (CF), and root mean square frequency (RMSF) of the power spectrum of time series ${\boldsymbol{D}}$ in the control group were significantly lower than those in the FEP group, while the root of variance frequency (RVF) of time series ${\boldsymbol{D}}$ in the control group was significantly larger than that in the FEP group (Fig. 5a–e). Using the separate template, we replicated the same result, and observed a further reduction in the p-value (Fig. 4e–h and Fig. 5f–j).

### Analyses for time series ${\boldsymbol{Z}}$

**Fig. 6:**
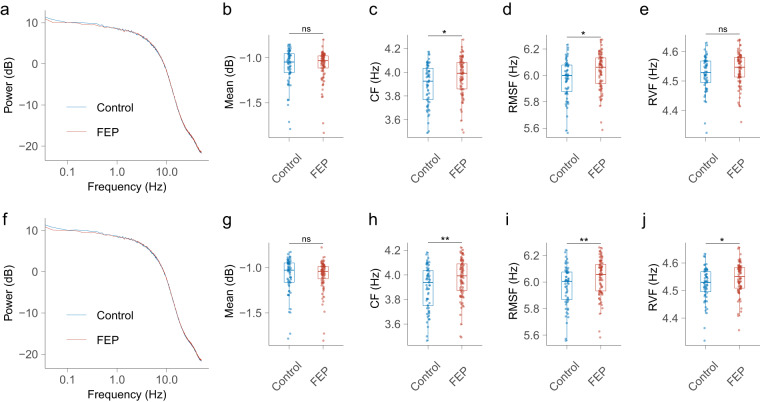
**The frequency domain characteristics of the complex time series** ***Z***. Using the shared template: (**a**) the power spectrum of time series ${\boldsymbol{Z}}$ in control and FEP groups. (**b**) comparison of the mean power between groups. (**c**) comparison of the CF between groups. (**d**) comparison of the RMSF between groups. (**e**) comparison of the RVF between groups. Using the separate template: (**f**) the power spectrum of time series ${\boldsymbol{Z}}$ in control and FEP groups. (**g**) comparison of the mean power between groups. (**h**) comparison of the CF between groups. (**i**) comparison of the RMSF between groups. (**j**) comparison of the RVF between groups. ns, non-significant; FEP, first-episode psychosis; CF, centre of frequency; RMSF, root mean square frequency; RVF, root of variance frequency.

Similarly, the control and FEP groups showed different oscillatory patterns of time series ${\boldsymbol{Z}}$ (Fig. 6a, f). Using the shared template, the CF and RMSF of the power spectrum of time series ${\boldsymbol{Z}}$ in the control group were significantly lower than those in the FEP group (Fig. 6c, d), while the difference of mean power and RVF were not significant between groups (Fig. 6b,e). Using the separate template, the difference in mean power continued to be non-significant (Fig. 6g). However, the differences in CF and RMSF remained significant, with a notable decrease in the p-value (Fig. 6h,i). Additionally, the RVF in the FEP group was observed to be significantly larger compared to that in the control group (Fig. 6j).

### Correlation analysis

**Fig. 7:**
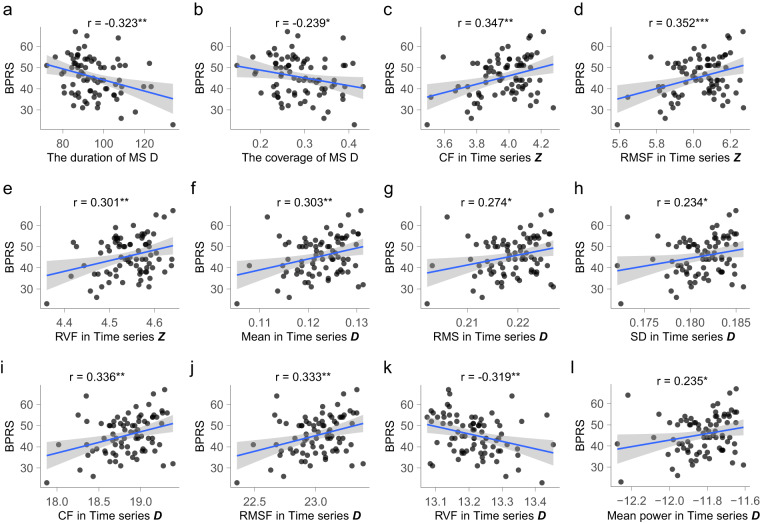
**Correlation analysis between microstate features and BPRS**. Using shared template: (**a**) correlation analysis between the duration of microstate D and BPRS. (**b**) correlation analysis between the coverage of microstate D and BPRS. (**c**) correlation analysis between the CF in time series ***Z*** and BPRS. (**d**) correlation analysis between the RMSF in time series ***Z*** and BPRS. (**e**) correlation analysis between the RVF in time series ***Z*** and BPRS. (**f**) correlation analysis between the mean distance in time series ***D*** and BPRS. (**g**) correlation analysis between the RMS in time series ***D*** and BPRS. (**h**) correlation analysis between the SD in time series ***D*** and BPRS. (**i**) correlation analysis between the CF in time series ***D*** and BPRS. (**j**) correlation analysis between the RMSF in time series ***D*** and BPRS. (**k**) correlation analysis between the RVF in time series ***D*** and BPRS. (**l**) correlation analysis between the mean power in time series ***D*** and BPRS. BPRS, Brief Psychiatric Rating Scale; CF, centre of frequency; RMSF, root mean square frequency; RVF, root of variance frequency. SD, standard deviation; RMS, root mean square.

Data on the Brief Psychiatric Rating Scale (BPRS) scores were unavailable for two patients. Using the shared template, we conducted correlation analyses between microstate features and the total BPRS score in FEP patients (Fig. 7). We found that the duration and coverage of microstate ***D****,* as well as the RVF in time series ***D***, exhibited a negative correlation with the total BPRS score. Conversely, the CF, RMSF, and RVF of the power spectrum in time series ***Z*** showed a positive correlation with the total BPRS score. Similar positive correlations were observed for the mean power, CF, and RMSF of the power spectrum in time series ***D*** with the BPRS total score. Additionally, the mean value, RMS, and SD in time series ***D*** also positively correlated with the BPRS. When applying the separate template, these mentioned correlations retained their significance (Supplementary Figure 4).

### Comparisons between medicated and medication-naïve patients

Medication details were unavailable for two patients. We observed no significant differences in any microstate features between medicated and medication-naïve patients, irrespective of whether the shared or separate templates were used (Supplementary Table 2 and Supplementary Figure 5–7).

### Machine learning

**Fig. 8:**
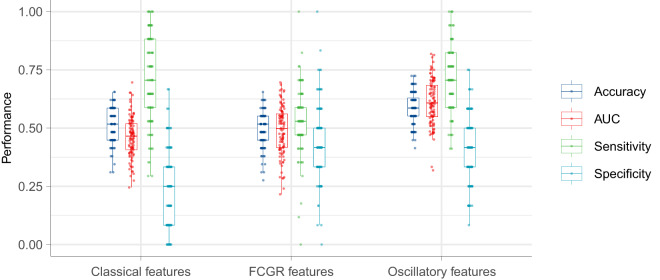
**The results of machine learning**. AUC, the area under the curve; CGR, chaos game representation; FCGR, frequency matrix CGR.

Employing classical microstate features resulted in a mean Area Under the Curve (AUC) value of 0.46. When using FCGR as features, the mean AUC value slightly increased to 0.49. However, the use of oscillatory features derived from the microstate CGR notably improved the mean AUC value to 0.61 (Fig. 8).

## Discussion

This study represents a pioneering exploration of the CGR approach in the analysis of microstate sequences, filling a notable gap in current research. By adopting this innovative perspective, we have uncovered previously unknown characteristics and significantly deepened our understanding of microstate sequences. We applied the CGR method to a publicly available dataset, and to ensure transparency and reproducibility, we have included the complete code used in our analysis. While we focused on the 4 canonical microstate classes, it is worth noting that the CGR approach can be readily adapted to accommodate varying numbers of microstate classes, differing only in the number of vertices. This flexibility enhances the applicability of CGR to diverse microstate analyses. Our key findings can be summarized as follows:

- CGR emerges as a promising tool for chronnectome fingerprinting, offering a visually compelling representation of microstate sequences by establishing a one-to-one correspondence between CGRs and microstate sequences.
- Time series ${\boldsymbol{D}}$ and ${\boldsymbol{Z}}$, obtained through microstate sequence CGR, offer a higher level of detail compared to conventional microstate features such as duration, occurrence, coverage, and transition probability. These fine-grained features provide a more comprehensive and nuanced understanding of the underlying dynamics and patterns within microstate sequences.
- Features derived from microstate CGR demonstrate potential as group-level neurobiomarkers for psychiatric disorders, while also enabling the identification of patients at an individual level.

The chronnectome refers to a description of time-varying functional connectivity^15^. Most chronnectome studies have used fMRI datasets; in particular, two studies have tried to construct chronnectome fingerprinting based on fMRI^16,17^. However, the low temporal resolution of fMRI does not allow it to reveal chronnectome characteristics on a fast time scale, which may lead to the loss of some useful information. Nonetheless, EEG microstates reflect underlying brain functional networks^2^; thus, EEG microstate sequences could represent time-varying functional networks. Each microstate sequence can be visualized by a unique CGR image; thus, this approach appears to be promising for chronnectome fingerprinting. Our results showed that some microstate subsequences were distinct between patients and controls. Previous studies have also consistently reported different subsequence patterns between patients and controls^9^. Microstate sequences have a long-term dependency but finite memory content^7^, RNN appears to be able to construct microstate sequences with high precision within a short period^10^, and sample entropy is reduced as the template length increases^9^. These results consistently indicated that these short repeatedly occurring subsequences, analogous to motifs in DNA, might be necessary for normal brain function in a resting state. Moreover, these short subsequences could be visualized easily by the corresponding cell in FCGR with different resolutions in the microstate sequence. However, although we identified these microstate subsequences, we did not determine their function. A previous study reported that traditional microstate features change during execution of cognitive tasks^18^. Thus, microstate subsequences may represent a period of coordination and cooperation among successive networks. We speculate that these short-repeated subsequences may be closely associated with specific cognitive functions, which could be visualized by the micrsotate FCGR. Further studies are needed to address this open question.

The four canonical microstates (A, B, C, and D) were consistently identified in each of the 100 iterations of microstate clustering, regardless of using the shared or separate templates for each group. This consistency underscores the robustness of these canonical microstates, aligning with previously established reliability^3^. In terms of traditional parameters, using the shared template revealed no significant differences between the FEP and control groups. However, using the separate template, it was observed that the duration of microstates A and D was significantly shorter, while microstates B and C occurred more frequently in FEP patients. These findings partially align with prior research. Murphy *et al*.^9^ reported a reduced duration of microstate A in early-course psychosis patients^9^, while Sun *et al*.^19^ observed increased duration, occurrence, and contribution of microstate C, and a decreased contribution and occurrence of microstate D in FEP patients^19^. Additionally, da Cruz *et al*.^20^ found an increased presence of microstate C and a decreased presence of microstate D in schizophrenia patients compared to controls^20^. Conversely, de Bock *et al*.^21^ identified an increased presence of microstate A and a decreased presence of microstate B in FEP patients^21^. The differences between the findings of de Bock *et al*.^21^ and those of Murphy *et al*.^9^, as well as our study, may be attributed to the use of only 19-channel EEG by de Bock *et al*.^21^, compared to the more than 32-channel EEG used in the other studies. Our study employed a 49-channel EEG for microstate analysis. Zhang *et al*.^22^ have indicated that microstate analysis becomes unreliable with fewer than 20 electrodes^22^. This might also explain the discrepancies in findings between medicated and medication-naïve patients; unlike a previous study using a 19-channel EEG^23^, our study found no significant differences.

Consistently, in both the shared and separate templates, a range of features from time series ***D*** and ***Z*** exhibited significant differences between the control group and the FEP group. This consistency underscores the potential of features derived from microstate sequence CGRs to provide more nuanced insights than traditional microstate features. This is reasonable because each CGR was constructed using a whole microstate sequence that contained relative positional information for each microstate and thus could be used as a fine-grained biomarker for neuropsychiatric disorders. Most microstate studies have focused on temporal characteristics, with few studies investigating the oscillatory features in microstate sequences. Our study filled this gap by exploring the power spectral properties of time series ${\boldsymbol{D}}$ and ${\boldsymbol{Z}}$ derived from the microstate sequence. A previous study using an information-theoretical approach demonstrated that microstate sequences have periodicity, and proposed that microstate sequences could inherit periodicity from EEG signals^7^. In our study, we observed a power spectral peak near 10 Hz in time series ${\boldsymbol{D}}$ derived from the microstate sequence. This phenomenon suggests that the time series ${\boldsymbol{D}}$ may share similar periodicity with the EEG signal and confirms that the microstate sequence also has oscillatory properties. Similar to previous findings that oscillatory patterns in microstate sequences are quite different between subjects^7^, the power spectral patterns of time series ${\boldsymbol{D}}$ and ${\boldsymbol{Z}}$ also showed substantial intersubject variability. Inspired by the findings of a previous study comparing DNA similarity^24^, the time series ${\boldsymbol{Z}}$ may serve as a functional personal identification. However, verification of this speculation is beyond the scope of this study, and further studies are needed to address this interesting issue.

The rationale behind clustering microstates using a shared template for all subjects was to simulate potential machine learning applications in a clinical setting. For a new subject, the choice of template for backfitting is not predetermined, as it is initially unclear whether the individual is a patient or a healthy control. A simple solution to this issue is to construct a shared microstate template to backfit EEG signals for future subjects regardless of whether she or he is a patient. We demonstrated the feasibility of this approach by obtaining acceptable GEVs when using a shared microstate template for both the patient group and the control group. Most AUCs we obtained were less than 70%; there are multiple possible explanations for this. First, microstate analyses cannot explain all global variance, and the GEV for all subjects was less than 85% and even less than 65% for a few subjects. It seems reasonable that the microstate sequence may not be reliable for subjects with a lower GEV. Second, we know that EEG is very noisy; thus, microstate sequences may also be affected by noise. This means that the majority of subsequences may be background noise, which could disturb a better classification performance. Developing a noise reduction approach for microstate sequences is needed for future research. Third, all EEG data we analysed in this study were collected in the resting states; generally, subjects may be thinking about various things when they are asked to keep a so-called “resting state”. These factors could increase heterogeneity between subjects. Notably, asking subjects to perform a specified task may reduce this heterogeneity. Overall, periodicity is a basic property of microstate sequences. Here, we demonstrated the potential capacity of microstate oscillatory features for individual patient classification.

There are some limitations of this study. First, only FEP patients were included in our study and our results may not be applicable to other neuropsychiatric disorders. Second, we only revealed features of microstate sequence CGRs in a resting state, and the characteristics of microstate sequence CGRs in a task state have not yet been elucidated. Based on our results, we could expect microstate CGRs to be different at different task states, specifically in terms of the FCGRs at a certain resolution, and to show a different oscillatory pattern. Third, although the application of machine learning showed some promising results, our sample size is small and these results were not validated in an independent dataset. It is also unclear whether different EEG acquisition modality (such as different numbers of channels) could affect the generalization of our results.

In conclusion, our study unveils the untapped potential of CGR in the analysis of microstate sequences, shedding new light on their characteristics. Our findings have significant implications for both the field of neurobiology and clinical practice, and our study may inspire further investigations in this promising area of research.

## Methods

### Data source

A public dataset on OpenNEURO was used in this study^25–27^. Briefly, 62 healthy controls and 81 patients with FEP were included in the dataset. The mean ages of the healthy controls and patients with FEP were 22.86 ± 4.71 years old and 22.73 ± 4.85 years old, respectively, which an independent sample test revealed to be not significantly different (t = 0.158, p = 0.874). There were 26 females and 36 males in the control group and 25 females and 56 males in the patient group. Although the ratio of males in the patient group was greater than that in the control group, the chi-square test did not show a significant difference between the groups (${\chi }^{2}$ = 1.876, p = 0.171). Resting EEG data were collected with the participant’s eyes open for 5 minutes using an Elekta Neuromag Vectorview system with a 60-channel cap.

### EEG data preprocessing

First, we downloaded all the relevant EEG data from the OpenNEURO website^28^. All data preprocessing was performed using the EEGLAB toolbox in MATLAB 2019a^29^. Because the original dataset consisted of two datasets, there are slight differences in the electrodes and sampling rate used. Most of the data were recorded with a sampling rate of 1000 Hz, but some were recorded with a sampling rate of 3000 Hz; these data were downsampled to 1000 Hz for consistency. As performing microstate analysis requires the same electrodes, we also selected the shared electrodes from the two original datasets for analysis. EEG data were filtered with a bandpass filter from 1 to 80 Hz and a notch filter at 60 Hz. Each segment of EEG data was inspected manually to detect bad channels and segments. Bad channels were interpolated using the spherical method, and bad segments were deleted before running independent component analysis (ICA). ICLabel was used to classify independent components and automatically remove artefact components^30^. Finally, all EEG data were rereferenced to an average reference.

### Microstate extraction

The Microstate toolbox was used for microstate extraction^31^. All EEG data, encompassing 49 channels, underwent low-pass filtering at 45 Hz and were subsequently downsampled to 100 Hz prior to conducting the microstate analysis. Initially, a group-level template was constructed using data from all subjects. We randomly selected 1000 global field power (GFP) peaks per subject and concatenated them before conducting modified k-means clustering. The number of random initializations of the modified k-mean was set to 100, and the maximum number of iterations was set to 1000. We ignored the polarity of the topographical map and selected four canonical microstates due to their well-established reliability^3^. Second, we used the template microstate prototypes for backfitting each subject’s EEG data. For temporal smoothing, a microstate with a duration of less than 30 ms was classified as the next most likely microstate class measured by global map dissimilarity (GMD)^31^. Third, classical dynamic characteristics (mean duration, coverage, and occurrence) were calculated. For each subject, the microstate sequence was extracted as a series of microstate labels at each time point before entering subsequent analyses. For robustness, we repeated the aforementioned procedure 100 times. To assess the potential impact of different templates on our results, we additionally created group-level microstate templates for the control group and the FEP group separately. Subsequently, we employed the control-template to backfit EEG data within the control group and the FEP-template for backfitting EEG data within the FEP group.

### CGR construction

We used the microstate sequence for CGR construction. The microstate sequence refers to a series of microstate labels for each time point, e.g., “AABBAADDD”. First, in a two-dimensional space, we set several vertices for the corresponding microstate classes and set the coordinate of the initial point to the centre. The corresponding coordinate of each microstate in the microstate sequence was defined as half the distance between the previous coordinate of the microstate and the vertex coordinate of the current microstate. Thus, for a given microstate sequence, the coordinate of each microstate (${P}_{n}$) in the CGR is given by:

$${P}_{n}=\frac{1}{2}\times \left({P}_{n-1}+V\right),n\in \left\{1,2,\ldots ,N\right\}$$

where $N$ is the length of a given microstate sequence, and ${P}_{0}$ is the coordinate of the initial point. $V$ is the vertex coordinate of the n-th microstate. A CGR illustration is described in Fig. 1.

### Data analyses

The analytical flowchart is described in Fig. 1. Since the length of the microstate sequence was different among subjects, we standardized the FCGR at a resolution of ${2}^{m}\times {2}^{m}$ by dividing by $\frac{N}{{2}^{m}\times {2}^{m}}$, where $N$ is the length of a given microstate sequence. FCGRs were constructed using the “kaos” package in R 4.1.0^14^.

Then, we defined a distance time series ${\boldsymbol{D}}$ as follows:

$${\boldsymbol{D}}=\{{{\boldsymbol{D}}}_{1},{{\boldsymbol{D}}}_{2},\ldots {{\boldsymbol{D}}}_{{\boldsymbol{n}}}\}\,,{{\boldsymbol{D}}}_{{\boldsymbol{n}}}=\sqrt{{({x}_{n}-{x}_{n-1})}^{2}+{({y}_{n}-{y}_{n-1})}^{2}}$$

where $({x}_{n,}{y}_{n})$ is the coordinate of ${P}_{n}$ in the CGR of the microstate sequence. ${D}_{n}$ is the Euclidean distance from the previous microstate to the current microstate. The MATLAB code used for this analysis was adapted from a previous study^24^.

Additionally, we defined a complex time series ${\boldsymbol{Z}}$ as follows:

$${\boldsymbol{Z}}=\left\{{{\boldsymbol{Z}}}_{1},{{\boldsymbol{Z}}}_{2},\ldots {{\boldsymbol{Z}}}_{{\boldsymbol{n}}}\right\},{{\boldsymbol{Z}}}_{{\boldsymbol{n}}}={x}_{n}+{y}_{n}i$$

where $({x}_{n,}{y}_{n})$ is the coordinate of ${P}_{n}$ in the CGR of the microstate sequence.

For both ${\boldsymbol{D}}$ and ${\boldsymbol{Z}}$, we performed a discrete Fourier transform (DFT) to transform them into the frequency domain and calculated their power spectrum. We calculated the mean power, CF, RMSF, and RVF for the power spectra of ${\boldsymbol{D}}$ and ${\boldsymbol{Z}}$. Additionally, we calculated the mean, SD, and RMS of ${\boldsymbol{D}}$. Independent t-tests were conducted between the control group and patient group, and multiple comparisons were corrected using the FDR. We also performed correlation analysis between microstate features and BPRS scores. Additionally, we conducted comparisons of microstate features between medicated patients and medication-naïve patients.

### Machine learning

To investigate the potential of the aforementioned features to differentiate patients with FEP from healthy controls, we partitioned 20% of the dataset as the test set and allocated 80% of the dataset as the training set. We conducted 5-fold cross-validation within the training set. A support vector machine (SVM) with a linear kernel was used as the model. To objectively assess model performance, we replicated the aforementioned procedure 100 times and computed the mean values of specificity, sensitivity, accuracy, and AUC. Machine learning was performed using Scikit-learn in Python 3.9^32^. Specifically, we assessed the following features: a) classical microstate features (duration, coverage, occurrence, and transition probabilities); b) oscillatory features derived from ***Z*** and ***D*** (mean power, CF, RMSF, RVF, mean, RMS, and SD); c) the FCGR of the microstate sequence. For machine learning, we exclusively utilized microstate features derived from the shared template.

### Supplementary information

Supplementary Information

## Data Availability

The datasets resulting from our analyses have been made publicly accessible on the Figshare website^33^. This dataset comprises four key components: a) The file titled “1. Preprocessed data” encompasses preprocessed EEG data. b) The file titled “2. Microstate_extraction” encompasses 100 microstate templates derived from all subjects, alongside 100 templates each from the control and FEP groups. c) The file titled “3. Microstate_sequence_CGR_features” contains microstate labeling text files for each subject, utilizing both shared and separate templates. d) The file titled “4. Figures_codes” encompasses the data employed in generating the figures presented in this study.

## Code availability

The code used in our analysis is available on GitHub (https://github.com/zddzxxsmile/Chaos-game-representation-of-EEG-microstate).
