# Supplementary material for: Changes in oscillatory patterns of microstate sequence in patients with first-episode psychosis
Source: Sci Data. 2024 Jan 5;11:38. doi: 10.1038/s41597-023-02892-8 (PMC10770397; doi:10.1038/s41597-023-02892-8)
Supplement: Supplementary file 1 — Supplementary Information [file 41597_2023_2892_MOESM1_ESM.pdf]

Supplementary information

Title	Page
**Supplementary Figure 1　The four microstate topographies in the shared template for 100 times.**	**2**
**Supplementary Figure 2　The four microstate topographies in the control group template for 100 times.**	**3**
**Supplementary Figure 3　The four microstate topographies in the FEP group template for 100 times.**	**4**
**Supplementary Table 1　Comparisions of microstate transition probatilities between FEP and control groups.**	**5**
**Supplementary Figure 4　Correlation analysis between microstate features and BPRS.**	**6**
**Supplementary Table 2　Comparisions of microstate transition probatilities between medicated patients and medication-naïve patients.**	**7**
**Supplementary Figure 5　Comparisons of classical microstate features between medicated patients and medication-naïve patients.**	**8**
**Supplementary Figure 6　Comparisons of microstate features derived from CGR between medicated patients and medication-naïve patients using the shared template.**	**8**
**Supplementary Figure 7　Comparisons of microstate features derived from CGR between medicated patients and medication-naïve patients using the separate template.**	**9**

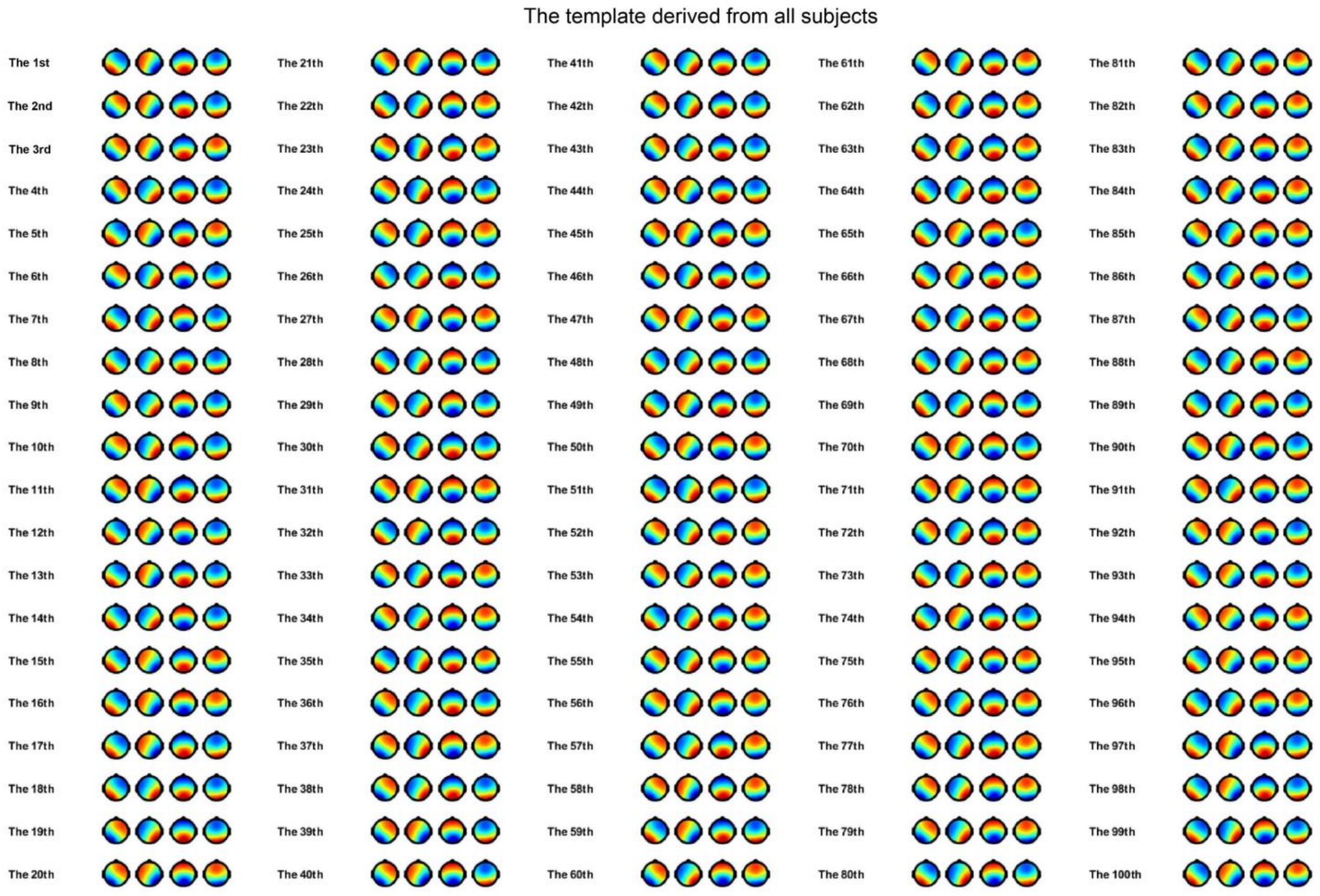

Supplementary Figure 1. The four microstate topographies in the shared template for 100 times.

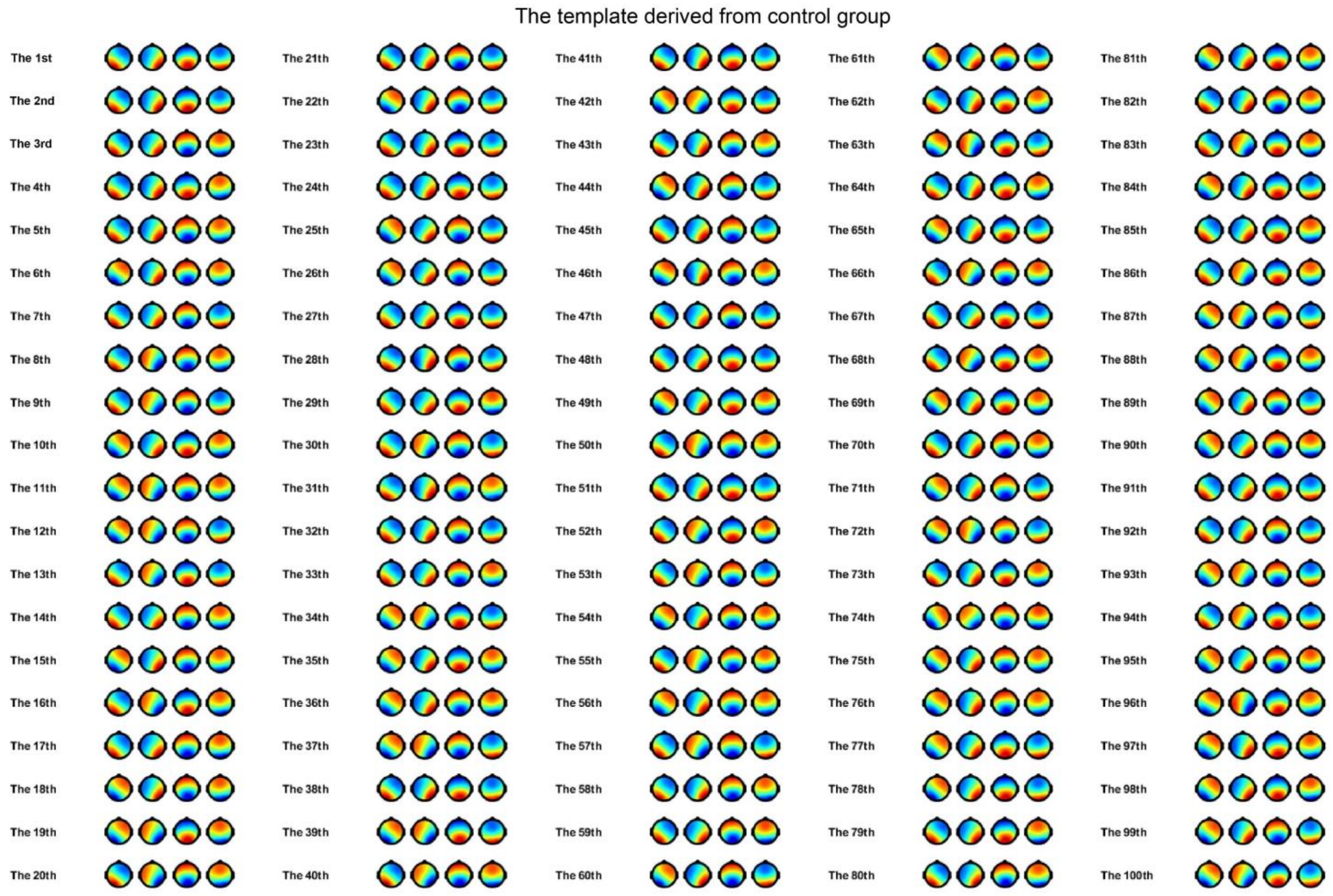

Supplementary Figure 2. The four microstate topographies in the control group template for 100 times.

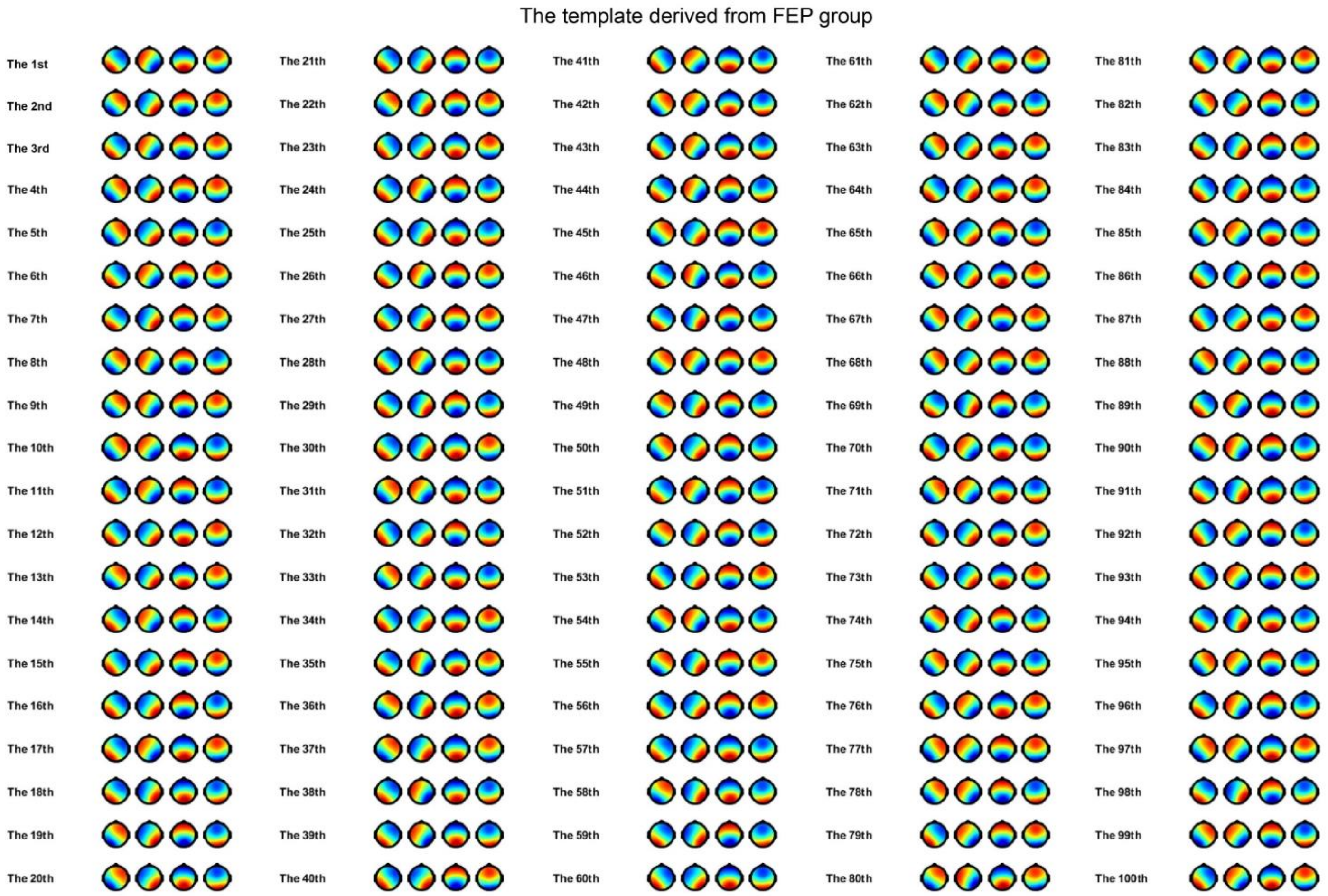

Supplementary Figure 3. The four microstate topographies in the FEP group template for 100 times.

	Control (N=61) Mean (SD)	FEP (N=81) Mean (SD)	t value	p value
Using shared template				
A→B	0.29 (0.06)	0.29 (0.06)	0.268	0.789
A→C	0.34 (0.06)	0.35 (0.07)	0.178	0.859
A→D	0.37 (0.07)	0.36 (0.06)	-0.450	0.653
B→A	0.31 (0.07)	0.31 (0.06)	0.270	0.788
B→C	0.33 (0.06)	0.34 (0.06)	0.189	0.850
B→D	0.36 (0.07)	0.35 (0.06)	-0.452	0.652
C→A	0.30 (0.06)	0.31 (0.05)	1.283	0.202
C→B	0.28 (0.05)	0.28 (0.05)	0.433	0.665
C→D	0.42 (0.09)	0.40 (0.08)	-1.139	0.257
D→A	0.31 (0.05)	0.32 (0.06)	0.825	0.411
D→B	0.29 (0.05)	0.29 (0.06)	0.329	0.743
D→C	0.40 (0.08)	0.39 (0.09)	-0.774	0.440
Using separate template				
A→B	0.29 (0.06)	0.30 (0.06)	0.964	0.337
A→C	0.34 (0.06)	0.35 (0.06)	1.252	0.213
A→D	0.38 (0.07)	0.35 (0.06)	-2.179	**0.031**
B→A	0.31 (0.07)	0.30 (0.06)	-1.269	0.207
B→C	0.33 (0.07)	0.35 (0.07)	2.246	**0.026**
B→D	0.36 (0.07)	0.35 (0.06)	-0.993	0.322
C→A	0.31 (0.06)	0.31 (0.05)	-0.511	0.610
C→B	0.28 (0.06)	0.30 (0.05)	2.311	**0.022**
C→D	0.41 (0.09)	0.40 (0.08)	-1.115	0.267
D→A	0.32 (0.06)	0.30 (0.06)	-2.079	**0.039**
D→B	0.29 (0.05)	0.30 (0.05)	1.199	0.232
D→C	0.39 (0.08)	0.40 (0.08)	0.659	0.511

Supplementary Table 1. Comparisions of microstate transition probatilities between FEP and control groups. FEP, first-episode psychosis; SD, standard deviation.

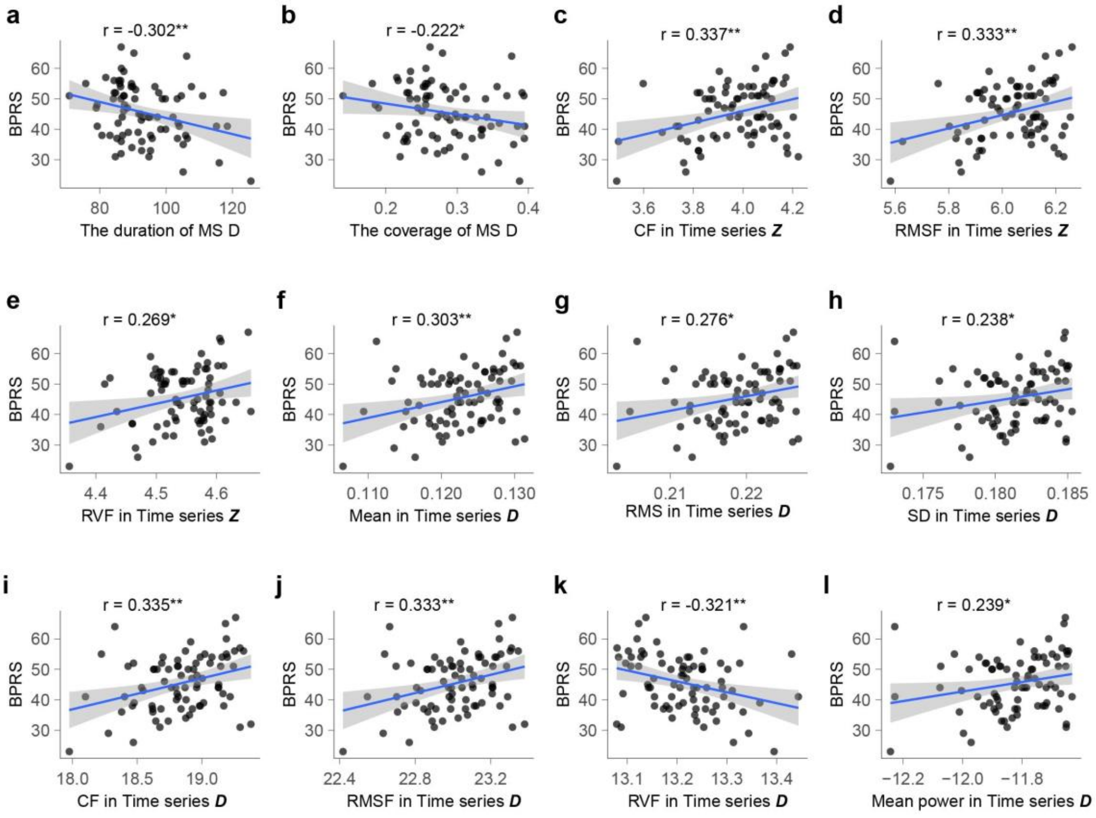

Supplementary Figure 4. Correlation analysis between microstate features and BPRS. Using separate template: a. correlation analysis between the duration of microstate D and BPRS. b. correlation analysis between the coverage of microstate D and BPRS. c. correlation analysis between the CF in time series ***Z*** and BPRS. d. correlation analysis between the RMSF in time series ***Z*** and BPRS. e. correlation analysis between the RVF in time series ***Z*** and BPRS. f. correlation analysis between the mean distance in time series ***D*** and BPRS. g. correlation analysis between the RMS in time series ***D*** and BPRS. h. correlation analysis between the SD in time series ***D*** and BPRS. i. correlation analysis between the CF in time series ***D*** and BPRS. j. correlation analysis between the RMSF in time series ***D*** and BPRS. k. correlation analysis between the RVF in time series ***D*** and BPRS. l. correlation analysis between the mean power in time series ***D*** and BPRS. BPRS, Brief Psychiatric Rating Scale; CF, centre of frequency; RMSF, root mean square frequency; RVF, root of variance frequency. SD, standard deviation; RMS, root mean square.

	With medication (N=54) Mean (SD)	Without medication (N=25) Mean (SD)	t value	p value
Using shared template				
A→B	0.30 (0.06)	0.28 (0.07)	1.614	0.111
A→C	0.34 (0.07)	0.36 (0.06)	-1.025	0.309
A→D	0.36 (0.05)	0.37 (0.07)	-0.560	0.577
B→A	0.31 (0.06)	0.31 (0.07)	-0.160	0.874
B→C	0.34 (0.07)	0.34 (0.06)	0.118	0.906
B→D	0.35 (0.06)	0.35 (0.08)	0.036	0.972
C→A	0.31 (0.05)	0.33 (0.07)	-1.130	0.262
C→B	0.29 (0.04)	0.27 (0.06)	1.451	0.151
C→D	0.40 (0.07)	0.40 (0.10)	-0.119	0.906
D→A	0.31 (0.05)	0.32 (0.06)	-0.823	0.413
D→B	0.30 (0.05)	0.28 (0.06)	1.508	0.136
D→C	0.39 (0.08)	0.39 (0.10)	-0.439	0.662
Using separate template				
A→B	0.30 (0.05)	0.28 (0.07)	1.858	0.067
A→C	0.35 (0.06)	0.36 (0.06)	-1.184	0.240
A→D	0.35 (0.05)	0.36 (0.07)	-0.644	0.521
B→A	0.30 (0.06)	0.30 (0.07)	-0.272	0.786
B→C	0.35 (0.07)	0.35 (0.06)	0.234	0.816
B→D	0.35 (0.06)	0.35 (0.07)	0.018	0.986
C→A	0.30 (0.05)	0.31 (0.07)	-0.987	0.327
C→B	0.30 (0.04)	0.29 (0.06)	1.350	0.181
C→D	0.40 (0.07)	0.40 (0.10)	-0.124	0.902
D→A	0.30 (0.05)	0.31 (0.06)	-0.693	0.490
D→B	0.31 (0.05)	0.29 (0.06)	1.637	0.106
D→C	0.39 (0.08)	0.41 (0.09)	-0.530	0.598

Supplementary Table 2. Comparisions of microstate transition probatilities between medicated patients and medication-naïve patients. FEP, first-episode psychosis; SD, standard deviation.

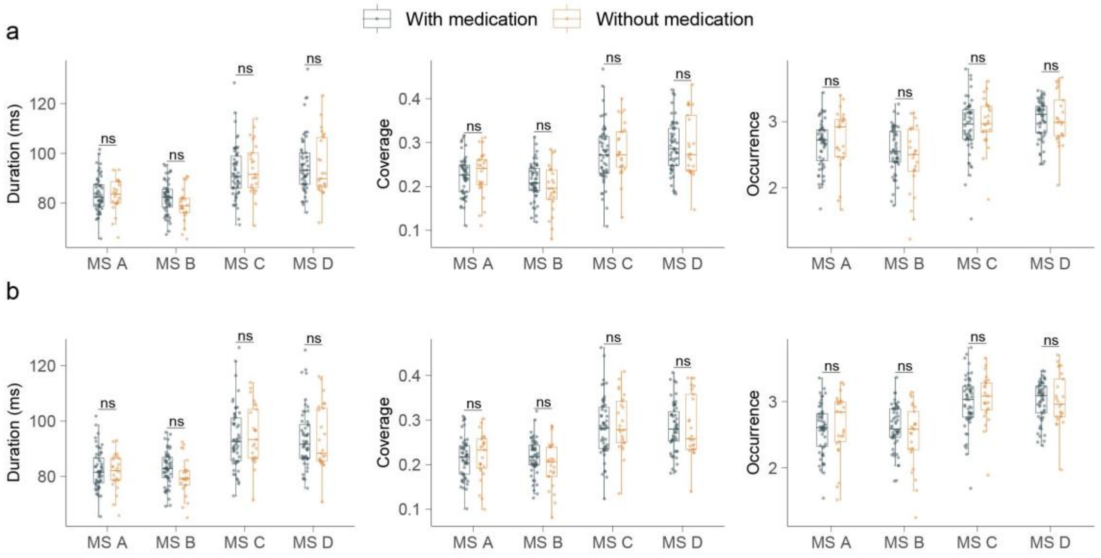

Supplementary Figure 5. Comparisons of classical microstate features between medicated patients and medication-naïve patients. a. using the shared template. b. using the separate template. ns, non-significant.

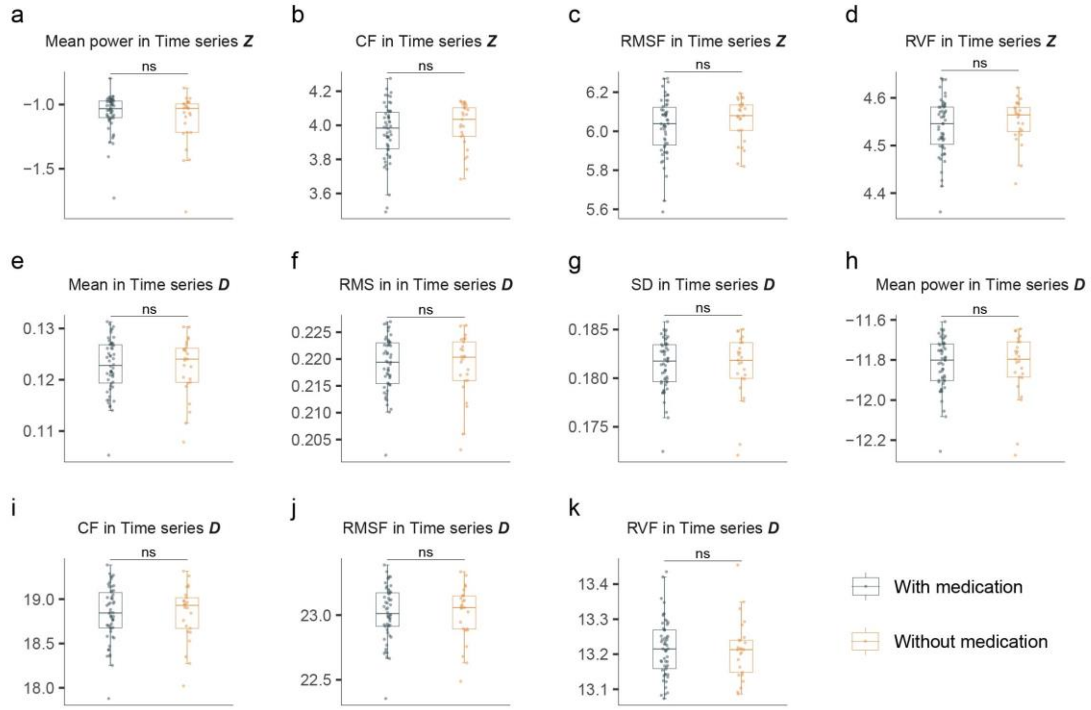

Supplementary Figure 6. Comparisons of microstate features derived from CGR between medicated patients and medication-naïve patients using the shared template. ns, non-significant; CF, centre of frequency; RMSF, root mean square frequency; RVF, root of variance frequency. SD, standard deviation; RMS, root mean square.

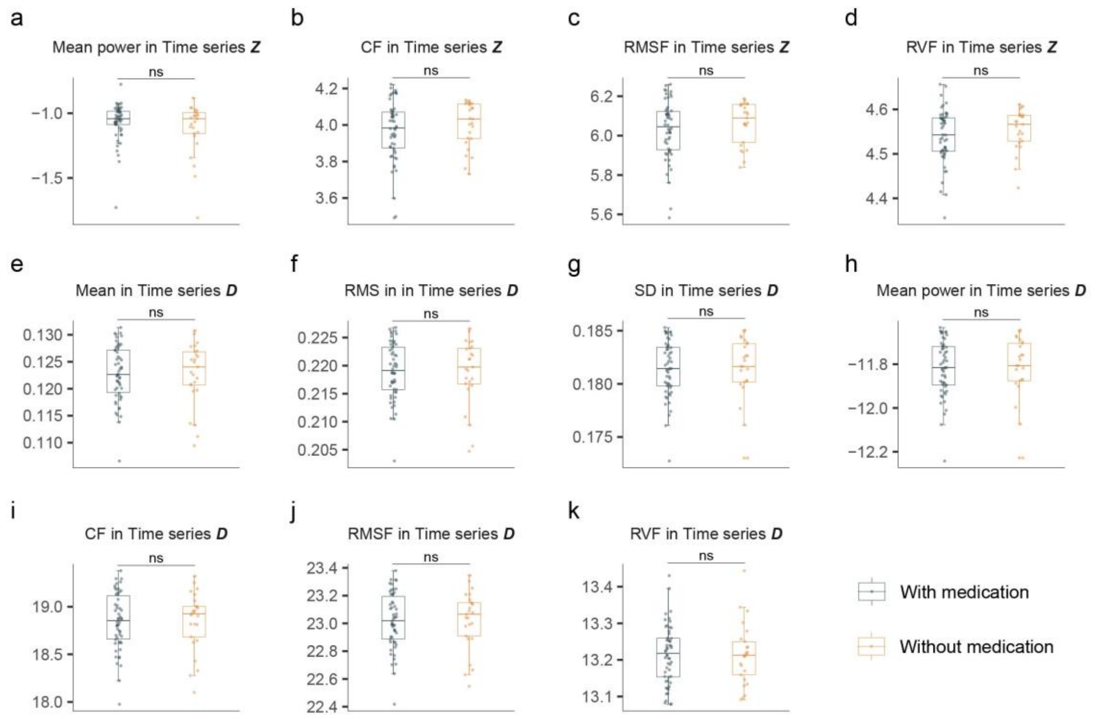

Supplementary Figure 7. Comparisons of microstate features derived from CGR between medicated patients and medication-naïve patients using the separate template. ns, non-significant; CF, centre of frequency; RMSF, root mean square frequency; RVF, root of variance frequency. SD, standard deviation; RMS, root mean square.
